# I Wouldn’t Even Want to Go There!

**DOI:** 10.1007/s40614-025-00440-w

**Published:** 2025-03-07

**Authors:** Mickey Keenan

**Affiliations:** School of Psychology, Ulster University, Coleraine, Northern Ireland

**Keywords:** ABA, Eclectic, Autism, Cultural differences, Ethics, Advocacy

## Abstract

Although applied behavior analysis (ABA) is regarded as providing the gold standard for interventions designed to meet the needs of autistic individuals in the United States, elsewhere this is not the case. In Northern Ireland, for example, successive governments have portrayed ABA simply as one of a number of commercially available interventions for autism. In this article, I argue that this view arises directly from the practice of behavior analysts who have courted the development of branded versions of ABA at the expense of promoting ABA directly. Because clinicians who advise government ministers are not trained in ABA, it is understandable that a discrimination issue arises whereby ministers are then encouraged not to invest in only “one of the commercially available interventions.” To address this problem, the article ends with a suggestion in how a specially designed ethical code of practice might hold behavior analysts accountable for the discrimination problems that could arise as a consequence of their actions in countries struggling to promote the uptake of ABA.

There is a well-known joke about a tourist in Ireland who asks one of the locals he meets for directions: “Excuse me, Sir,” he says, “could you possibly give me directions to Dublin?” The man replies: “Well sir, if I were you, I wouldn’t start from here.” Being Irish, I am exercising what I consider to be my birthright to relay my delight in the surrealist twist in the logic of that joke! In the context of this comment, I consider it a valuable starting point for addressing numerous issues around eclecticism and applied behavior analysis (ABA).

In a far-off land, a town called ABA is populated by many professionals who have a basic training in the natural science of behavior analysis. Many of these professionals have set up their own business and make a decent living out of it by providing services to those who value the effectiveness of the science from which the business is derived. These professionals have congregated in the suburbs of ABA, in what might be described as the Eclectic Valley of ABA. They are easily located using digital maps on various mobile devices. They are found on, for example, PBS street, PECS street, EIBI street, and so on.

The mix of services available in Eclectic Valley all stem from the work of dedicated teachers of the applied branch of the basic science. That much they have in common. The free market culture in which they thrive boasts a regular upturn in the number of businesses joining the valley every year. Within their culture, and in the absence of a dedicated national health service, businesses providing services for a variety of disabilities have attracted venture capitalists who occasionally soar above the valley searching for opportunities to make a financial investment that will ensure a good return on their money.

Some of the people from Eclectic Valley have at times noticed opportunities to expand their business into other countries where there are no towns like ABA (Keenan et al., 2010). Although financial opportunities beckon them, there are no regular townhall meetings dedicated to a collective assessment of issues that might arise from embedding their services within the cultural practices of other countries. Ethical considerations of cultural differences and discussions about how to manage the dialogue with public officials within respective health and social services of different communities has not been on the agenda. Instead, a wild-west mentality is exported whereby the promotion of training in ABA in a country takes a back seat to the promotion of specific interests cultivated in Eclectic Valley.

To take an example, I live in Northern Ireland, a country torn apart by years of community violence, and slowly struggling to get on its feet. In the midst of this recovery, there have been attempts to build a town like ABA. However, consequences produced by the actions of residents from Eclectic Valley continue to stymie these attempts. For example, not knowing the difference between ABA and Eclectic Valley, an education minister is on record as saying that ABA is “one of a number of commercially available treatments for autism” (Keenan, 2017a). Putting ABA in the same category as Eclectic Valley is something that easily happens when the various streets in Eclectic Valley are inadequately identified as being in a suburb of ABA in the tourist brochure from that far-off land. Unfortunately, there is no body to oversee the design of this brochure. Another example of the actions of a resident from Eclectic Valley clearly indicates the challenge for locals in other countries to build their version of a town like ABA. I remember a business from one street (that shall remain anonymous, but they know who they are) that even explicitly declined an invitation to ensure that the government in one European country was corrected in its depiction of the relation between “. . . Street” and ABA for fear of losing business if their association with ABA was to become public knowledge!

Some time ago, I submitted a Freedom of Information Request in my country, which asked for scientific evidence that the effectiveness of an eclectic approach to interventions for autism was equal to, or superior to, ABA (Keenan, 2017a). The government admitted that they didn’t have any evidence, yet they insisted that government policy would focus on adopting an eclectic approach. This might appear astounding if it wasn’t for the fact that the position on ABA as being only “one of a number of commercially available treatments for autism” has taken root. “Every child is different, and you can’t invest in only one thing for everybody” is the current mantra. Among the other treatments being referred to in this eclectic mix are PBS, PECS, etc.—all residents of Eclectic Valley. As a consequence of this category error, university programs designed to help produce professionals who might acquire skills in ABA are threatened.

These examples show how the term “eclectic” plays out in one country outside of the bubble of ABA town in that far-off land. It is sad that ethical concerns often take second place to business opportunities elsewhere as well. In the Czech Republic, for example, huge gains have been made to lift the profile of efforts to build a version of an ABA town (Gandalovičová, 2016). After much groundwork by staff from the local university, along with parents in the community, the government agreed to invest in setting up a masters’ course for professionals who could acquire the skills needed to build their ABA town. At the same time, the largest health insurance company in the country paid for the translation of an online course developed in Northern Ireland, SimpleSteps (https://simplestepsautism.com), which provides free online multimedia resources for training in behavior analysis. Things have been slow given the monumental task embarked upon by the small number of professionals who provide university training and who translate books for the community. All this work culminated in the Czech Republic being the first country in Europe to introduce a law recognizing the profession of behavior analyst. In recent years, though, the sound of a bugle from Eclectic Valley has entered the fray. PBS services offered by private organizations have moved in, with the result that a split in the community is beginning to emerge, mostly because seeds of anti-ABA rhetoric are being sown (see also the warning signs about the impact of bias against behavior modification from Woolfolk et al., 1977). An eclectic mix of interventions, à la Northern Ireland, might be viewed as a good thing by politicians. However, this is not the case when it dilutes the knowledge and skills needed to build an ABA town (Green, 2018). In addition, extortionate fees are charged for some of these short courses, often costing more than the only masters’ level training course in the country. As with many a good sales pitch, if a short course is more expensive, then it must be better! Of course, the fact that PBS Street is merely a suburb in ABA town is something that the consumers never hear.

In matters concerning quality in the teaching of behavior analysis, the words of Carol Pilgrim (2018) are relevant here:

. . . “to be” a behavior analyst entails a complex and inter-related repertoire sometimes described as a “world view”—one that is brought to bear continuously, not just when performing specific employment responsibilities. . . . Whether the student’s stated goals are to become an applied behavior analyst, a practicing BCBA, a research scientist, a scholar, or simply to pick up an undergraduate minor, my goal is to shape a behavior analyst—period, no qualifiers—to the fullest extent possible, in the time that I have with that student. (p. 204)

Unfortunately, the professionals at the university in the Czech Republic are now faced with an additional burden of learning how to manage the consequences of Brandolini’s law: “The bullshit asymmetry: the amount of energy needed to refute bullshit is an order of magnitude bigger than to produce it.”

Drawing from lessons in a number of European countries, Keenan et al. (2010) asked whether it is the case that inhabitants from Eclectic Valley who prioritize the promotion of branded versions of autism interventions don’t see the need to promote ABA per se. If they don’t see the need to promote ABA, then why should policy makers in another country care about investing in ABA? Indeed, why should those who obstruct the uptake of ABA in a community care about correcting the kind of category mistake prevalent in Northern Ireland and elsewhere when it is behavior analysts who helped to create the mistake in the first place? Keenan et al. (2010) proposed a simple solution to address the confusion created by residents of Eclectic Valley: “If there is a common goal to promote the development of Applied Behavior Analysis in other countries, then we suggest here some behavior in the form of a declaration that would help to make this happen” (pp. 140–141). Keenan (2017b) suggested that a list of signatories to a declaration that publicly anchors the heritage of their commercial products in ABA would be extremely helpful. The home of this declaration could be the Association for Behavior Analysis International (ABAI; https://www.abainternational.org/welcome.aspx). Such a declaration would be especially useful given the ethical landscape in the wake of the Behavior Analyst Certification Board’s (BACB) withdrawal from international certification (BACB, 2019). As stated in the BACB (2020) code of ethics: “The BACB does not have separate jurisdiction over organizations or corporations” (p. 3). This clearly implies that organizations providing ABA services are free to develop their own ethical code of practice. But it is unclear what happens if a board certified behavior analyst (BCBA) works in one of these organizations that does not uphold the ethical code outlined by the BACB: “Behavior analysts work to maximize benefits and do no harm by: . . . Focusing on the short- and long-term effects of their professional activities” (p. 4). This professional might have to comply with the ethical code of the organization that employs them, or else lose their job. If this ethical code is in conflict with that outlined by the BACB, they can be sanctioned by the BACB for choosing to follow the ethical code of the organization in preference to BACB code.

When geographical boundaries are factored into the picture, it is entirely possible for this scenario to be more than a simple word salad. When the BACB pulled out of its worldwide reach, many BCBAs had the feeling of losing a home. At this point, a different bugle sounded from Eclectic Valley, one that offered hope for those who felt isolated and stranded in hostile territory, i.e., their own country. “A nice little money earner” it could be said, by an organization that does not confer any legal legitimacy for practicing ABA within the home countries of its members. A different solution is to embolden isolated professionals to help set up their own ABA bodies so that they can eventually acquire professional recognition. This has been done in the UK (https://uk-sba.org/about-uk-sba/) and France (see in Nursel Ozkan’s overview of developments in France in Keenan et al., 2022). To help those starting from scratch, a funded European Erasmus+ project (https://euroba.org) provides many of the basic documents needed (at no cost), documents that can be adapted to meet requirements specific to individual countries while at the same time retaining a core body of material that can be used to generate a European wide community of behavior analysts.

Although many residents of Eclectic Valley offer services/training in other countries, there is no talk of an ethical code of practice that references the social consequences that arise from actions of a commercial entity. As indicated earlier, there is clear evidence that a professional with commercial interests can be averse to correcting the mistakes arising from their involvement in a country where training in ABA is not yet established. At the individual level, the BACB ethical guidelines emphasize the importance of conducting a functional analysis/assessment before implementing an intervention. Why does this level of protection not also apply to the actions of those who are ignorant of the consequences of their actions in another country? These problems can encompass and enshrine potential social disadvantage when only wealthy parents can pay for services from a private organization in a country where there are no existing ABA-based autism services. Where the misinformation about ABA from PBS providers is rampant (Stalford et al., 2024), point me to the document signed by residents of Eclectic Valley that identifies resolutions to ensure that malpractice like this should not occur. When the neurodiversity movement maligns ABA and confuses the applied science with questionable actions of individuals, where is the key document signed by residents of Eclectic Valley correcting the misinformation so as to ensure that the vulnerable children and families we serve are not disadvantaged (Dillenburger & Keenan, 2023; Graber & Graber, 2023). When vested interests in tribunals block access to behavior-analytic services (e.g., Blakemore-Brown, 2021; Byrne & Byrne, 2005; Keenan & Dillenburger, 2023), where are the bugles sounding alarm from Eclectic Valley? Let’s also not forget the consequences of the inaction of those who take advantage of jobs created by the pioneering work of people like Professor Lorri Unumb (2013) and others (Keenan et al., 2022). As the well-known warning against complacency, commonly attributed to 18th-century Irish philosopher Edmund Burke, says “The only thing necessary for the triumph of evil is for good men to do nothing.”

Ethical dilemmas in far distant lands are not the concern of U.S.-centric organizations. Who, for instance, provides consequences for fabricating damage to the growth of ABA in another country? Certainly not the organizations from Eclectic Valley where the bottom-line result of their work is measured in something other than the spread of the science from which they were born. I look forward to reading the minutes of the regular meetings in their townhall where an item on the standing agenda is the international consequences of their collective action. A politician asks one of the professionals he meets in Eclectic Valley for directions: “Excuse me, Sir,” he says, “could you possibly give me directions to ABA town?” The professional replies: “Well sir, if I were you, I wouldn’t even want to go there!”

## References

[CR1] Behavior Analyst Certification Board. (2019). BACB international development & support. https://www.bacb.com/international-development/. Accessed 1 Mar 2025.

[CR2] Behavior Analyst Certification Board. (2020). *Ethics code for behavior analysts*. https://www.bacb.com/wp-content/uploads/2022/01/Ethics-Code-for-Behavior-Analysts-240830-a.pdf. Accessed 1 Mar 2025.

[CR3] Blakemore-Brown, S. (2021). The long road: Defending the rights of children on the autism spectrum for access to the science of ABA in the UK: In the community, the curriculum and the courtroom. https://www.youtube.com/watch?v=BZN-ZJWDePs&t=1317s. Accessed 1 Mar 2025.

[CR4] Byrne, H., & Byrne, T. (2005). Mikey—Dealing with courts, tribunals and politicians. In M. Keenan, M. Henderson, K. Dillenburger, & K. P. Kerr (Eds.), *Applied behaviour analysis and autism: Building a future together* (pp. 208–218). Jessica Kingsley.

[CR5] Dillenburger, K., & Keenan, M. (2023). Autism and behavior analysis: From dissonance to dialogue. *International Electronic Journal of Elementary Education,**15*(3), 199–208.

[CR6] Gandalovičová, J. (2016). The arrival of ABA in the Czech Republic. *European Association for**Behaviour**Analysis Newsletter. *https://www.europeanaba.org/about-us/newsletter-archive. Accessed 1 Mar 2025.

[CR7] Graber, A., & Graber, J. (2023). Applied behavior analysis and the abolitionist neurodiversity critique: An ethical analysis. *Behavior Analysis in Practice,**16*, 921–937. 10.1007/s40617-023-00780-6 10.1007/s40617-023-00780-6 PMC9979895 37363652

[CR8] Green, G. (2018). Consumer corner: Identifying applied behavior analysis Interventions. *Science in Autism Treatment,**15*(2), 6. https://asatonline.org/research-treatment/resources/topical-articles/identifying-applied-behavior-analysis-interventions/. Accessed 1 Mar 2025

[CR9] Keenan, M. (2017a). *Evidence and policy: How to help families of children diagnosed with autism in Northern Ireland*. Stormont: KESS presentation. https://niassembly.tv/video/evidence-policy-help-families-children-diagnosed-autism-northern-ireland/. Accessed 1 Mar 2025.

[CR10] Keenan, M. (2017b). The fuzzy outline of an operant. *The Behavior Analyst,**40*, 187–191. 31976952 10.1007/s40614-017-0097-6 PMC6701214

[CR11] Keenan, M., & Dillenburger, K. (2023). Advocacy and open science in the UK: Case studies in the autism wars. *Behavior Analysis in Practice. *10.1007/s40617-023-00881-2 10.1007/s40617-023-00881-2 PMC12209167 40606424

[CR12] Keenan, M., Dillenburger, K., Moderato, P., & Röttgers, H.-R. (2010). Science for sale in a free market economy: But at what price? ABA and the treatment of autism in Europe. *Behavior and Social Issues,**19*, 126–143.

[CR13] Keenan, M., Dillenburger, K., Konrad, M-H., Debetencourt, N., Vuksan, R., Kourea, L., Ozkan, N., Abdelnour, H., Da Costa-Merenda, M., Pancocha, K., Kingsdorf, S., Schuldt, S., Mellon, R., Herman, A., Tennyson, A., Ayvazo, S., Moderato, P., Coppini, N., Schenk, J., Budzinska, A., ...., & Strömberg, D. (2022). Professional development of behavior analysts in Europe: A snapshot for 21 countries. *Behavior Analysis in Practice, 16,* 709–729. 10.1007/s40617-022-00754-0 10.1007/s40617-022-00754-0 PMC9648995 36406141

[CR14] Pilgrim, C. (2018). Some thoughts on shaping future behavior analysts: A call to stay true to our roots. *Behavior Analysis in Practice,**11*, 204–205. 10.1007/s40617-018-0233-0 30363845 10.1007/s40617-018-0233-0 PMC6182855

[CR15] Stalford, D., Graham, S., & Keenan, M. (2024). A discussion of positive behavior support and applied behavior analysis in the context of autism spectrum disorder in the UK and Ireland. *Behavior Analysis in Practice,**17*, 442–455. 10.1007/s40617-023-00905-x 38966271 10.1007/s40617-023-00905-x PMC11219608

[CR16] Unumb, L. (2013). *Keynote address by Dr Lorri Unumb at Centre for Behaviour Analysis (QUB) conference*. Queen’s University Belfast. https://www.youtube.com/watch?v=eGL7c0hCNlY. Accessed 1 Mar 2025.

[CR17] Woolfolk, A. E., Woolfook, R. L., & Wilson, G. T. (1977). A rose by any other name …: Labeling bias and attitudes toward behavior modification. *Journal of Consulting & Clinical Psychology,**45*(2), 184–191. 850002 10.1037//0022-006x.45.2.184

